# Signal Waveform Detection with Statistical Automaton for Internet and Web Service Streaming

**DOI:** 10.1155/2014/647216

**Published:** 2014-06-16

**Authors:** Kuo-Kun Tseng, Yuzhu Ji, Yiming Liu, Nai-Lun Huang, Fufu Zeng, Fang-Ying Lin

**Affiliations:** ^1^Harbin Institute of Technology, Shenzhen Graduate School, Shenzhen, China; ^2^Chunghwa Telecom Laboratories, Yangmei, Taoyuan 32601, Taiwan; ^3^Bartlett School, University College London, London WC1E 6BT, UK

## Abstract

In recent years, many approaches have been suggested for Internet and web streaming detection. In this paper, we propose an approach to signal waveform detection for Internet and web streaming, with novel statistical automatons. The system records network connections over a period of time to form a signal waveform and compute suspicious characteristics of the waveform. Network streaming according to these selected waveform features by our newly designed Aho-Corasick (AC) automatons can be classified. We developed two versions, that is, basic AC and advanced AC-histogram waveform automata, and conducted comprehensive experimentation. The results confirm that our approach is feasible and suitable for deployment.

## 1. Introduction

With the development of the Internet and web service computing and as more transactions are carried out online [1, 2], there is greater vulnerability to network attacks. Thus, Internet and web service content analyses have become important pieces of technology to protect our network security. Such systems collect information through a number of key points in the computer network or system and analyze it to determine whether there are the violations of the security policy or intrusion phenomenon.

Conventional packet analysis systems used packet classification and deep packet inspection, which may not be sufficient to detect all kinds of network service. Therefore, we have tried a new approach to analyze the network packets. We propose a new signal waveform based on deep packet classification. In this paper, we review some previous researches and discuss the existed approaches of packet analysis and then propose new analysis architecture based on a mathematical statistical approach. Compared with conventional methods, the network data within a time period is recorded to obtain the training waveform. The attention is on the statistical data of the waveform features, and we use their feature extractions as input to the classifiers.

Packet classification can be divided into two parts, training and testing. The training part is used to establish patterns and behavioural rules, and the testing part is for the classification and determination of the samples. In the training stage, data frame length information is extracted, and the integer of the length information of the data frame converted to a string. Then a certain number of characters are taken as a unit of the recording pattern and the pattern mode and its number of occurrences will be counted (the pattern retreats one packet each time). Through the training process, the system determines the signal waveform and its number of occurrences. Then, the same transform is carried out on other data, by loading the test string data into the Aho-Corasick (AC) automaton for matching, obtaining a match situation, and then we make a comparison with standard data. We use their differences as the judgment basis.

On the other hand, recent intrusion detection systems commonly use two detection technologies, namely, misuse detection and anomaly detection. Anomaly intrusion detection is a behaviour-based detection regime. Its basic principle is to determine whether a deviation from the so-called normal behaviour contour exists by comparing the current system and user's behaviour with the established system or user's normal behaviour contours, which can determine whether it is abnormal (intrusion) behavior; this is an indirect detection method. Misuse intrusion detection has also been referred to as knowledge-based detection, the principle of which is conducting attack feature extraction with known attack methods to constitute a signature database then processing pattern matching between the audit data and the feature library to determine the intrusion behaviour. This approach, which is based on the principles of the above two intrusion detection methods, consolidates the user's behaviour and methods of attacks and will put forward new intrusion detection principles for the whitelist and blacklist of network's behavior. That will enhance the efficiency of traditional anomaly detection and misuse detection methods.

The method applied in this paper is similar to misuse detection, in that the characteristics and rules of the packets are obtained for different kinds of applications, which will form the basis of the classification. This paper puts forward a new idea for packet detection by calculating the features of the network packet, which means that it draws on the allied technology of packet classification and uses these technologies as its foundation. However, this topic uses a certain flow of data packets as the object of the analysis and processing and extracts and calculates the signal waveform information of the characteristics to classify the data packets, which is different from the majority of packet classification algorithms based on a single or data connection packet. Furthermore, it implements a network-based real-time packet detection system, to carry out the detection and analysis of the network behaviour of the user. This paper with calculation and matching of the overall data packets flowing through the network can get more accurate grasp and analysis of user behaviour, as well as achieving the purpose of detection of user behaviour of network applications. At the same time, the algorithm can also be used for the detection of some forms of intrusion. In addition, through appropriate training, it can also be implemented in user identification and system safety rating.

In this paper, some related knowledge is introduced in Section 2, and our system architecture and proposed approaches are presented in Sections 3 and 4. In Section 5, we introduce some advanced analysis of this issue. And we have conducted some preliminary assessment of the approach, which will be described in Section 6. In Section 7, we conclude the paper and offer a look towards planned future work.

## 2. Background and Related Work

As effective means of network monitoring and traffic control, network packet classification technology has developed by leaps and bounds since its birth. At the same time, various classification methods and means have continually emerged, which have been used in a wide range of network packet inspection methods. In this section, an introduction and analysis of several classic packet classifications are given, as well as a brief summary of the research situation regarding the packet classification algorithms.

**Figure 1:**
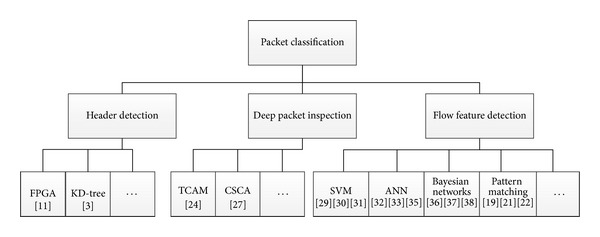
Categories of packet classification algorithms.

According to the different division standards, there are several different classifications of intrusion detection systems (see Figure 1).

Recent packet classification technology mainly includes Deep Packet Inspection method (DPI), Stateful Packet Inspection Method (SPI) (header packet classification), and inspections based on the flow characteristics of known data sets, such as support vector machines (SVMs) for classification and the artificial neural network classification algorithm, as well as the pattern matching classification algorithm.

DPI technology generally utilizes the packet payload to identify applications. DPI checks not only the header information, but also the deep packet payload content to determine the application layer protocol. DPI technology utilizes exact string on behalf of the network application mode or regular expressions for matching with the data packet payload, so that it can be determined whether the detected pattern is present in the data packet payload.

SPI is designed to prevent harmful or unsolicited packets entering the computer. SPI was originally developed for firewalls, but so far it has become an important factor in network-based intrusion detection systems (NIDS) and many other applications [3].

The so-called “state” denotes “things before recording.” Essentially, SPI is a dynamic packet filter working in a network layer. It checks each IP packet to obtain the necessary information and then the tracking network connection state, thus allowing or denying the subsequent traffic through the firewall. SPI gradually replaced the static packet filter and became an industry standard for network firewall solutions.

SPI tracks network connection states (such as TCP streams or UDP communications) and saves the important attributes of each connection into its memory. These attributes are collectively referred to as the state of the connection, which may include the IP address, port number, and serial number. State inspection monitors packet data and the connection status of incoming and outgoing packets and stores them into a dynamic state table. The accumulated data will be evaluated, and filtering decisions are based not only on predefined rules set by the administrator, but also on the configuration established by previous connections and packets belonging to similar connections.

On the other hand, many scholars have designed packet classifications and algorithms according to the known data set, such as the classification algorithm based on support vector machines (SVM-based) and artificial neural networks (ANN-based), as well as classification algorithm based on Bayesian network, calculation of pattern matching, and so forth.

The SVM is a supervised learning method that can be used for classification and regression analysis. It will map the training vectors to a high dimensional feature space, and each vector will be marked by its category. The SVM treats the classification as a quadratic optimization problem. The combination of SVM and generalization control technology, which can avoid the “curse of dimensionality” by setting the maximum interval between the different categories, makes it a practical tool in processing large number of dynamic collected data. By defining a series of SVMs, SVM constructs separating hyperplanes in the feature space to conduct data classification [4].

In the past, several research groups have proposed a variety of intrusion detection methods based on SVM technology. Fugate and Gattiker [5] proposed an anomaly detection method using SVM. Kim and Park [6] proposed a method of applying SVMs to network-based intrusion detection systems. Xie et al. [7] proposed a feature extraction algorithm using a factor analysis (FA) and support vector decision function ranking method (SVDFRM). Mukkamala et al. [8] described approaches to intrusion detection using SVMs. They built efficient and accurate classifiers to detect intrusions. Hu et al. [9] have proposed a host-based anomaly detection method using RSVM. Kim et al. [10] proposed a fusion of a genetic algorithm (GA) and SVM for anomaly detection in an intrusion detection system (IDS).

Artificial neural networks (ANN), also called neural networks, are a mathematical or computational model that imitates the structure and function of a biological neural network. The neural network is constitutive of interconnected artificial neurons, which uses the link method to calculate and process information. In most instances, the neural network is an adaptive system, which means that it can change its structure in the learning stage. So far, ANNs have been widely applied in the field of intrusion detection. Cannady [11] presented an approach to the process of misuse detection that utilizes the analytical strengths of neural network. Lippmann and Cunningham [12] improved high false-alarm rates by using a combination of discriminative training and generic keywords. Bivens et al. [13] used a time-window method. The method allows us to generalize input further than Cannady's method, enabling us to recognize longer multipacket attacks. They collect and monitor network traffic trends. Lorenzo-Fonseca et al. [14] proposed a sort of compromise between both ends of the scale: a model based on principal component analysis (PCA) as the chosen algorithm for reducing characteristics in order to maintain efficiency without hindering the capacity of detection. Moradi and Zulkernine [15] aimed to solve a multiclass problem in which the type of attack is also detected by the neural network. Wang et al. [16] proposed a novel approach for ANN-based IDS, the FC-ANN, to enhance detection precision for low-frequent attacks and detection stability.

The Bayesian network, also called evidential network, is an expansion of the Bayesian method and one of the most efficient models in the uncertain knowledge and reasoning field. It can be used to express and analyze uncertainty and probability events and conditionally relies on decision making with a variety of control factors, conducting reasoning from incomplete, imprecise, or uncertain knowledge or information [17, 18].

Similarly, there are many studies on Bayesian-based IDS. Bulatovic and Velasevic [19] suggested a Bayesian alarm network to work as an independent network intrusion detection agent. Kruegel et al. [20] utilized a Bayesian decision process to classify input events. Burroughs et al. [21] used Bayesian multiple hypothesis tracking as a basis for identifying the activities of individual attackers as they move across many networks. They improved the understanding of the attackers' behaviour by using existing data being gathered from distributed IDS. Cemerlic et al. [22] proposed an adaptive network intrusion detection system using a Bayesian network, trained with a mixed dataset containing real-world and DARPA dataset traffic.

At present, the use of a pattern matching method to detect intrusion is gradually becoming the mainstream of intrusion detection systems. As the core of pattern matching methods, string matching algorithms have been researched and applied widely. With the present development of pattern matching algorithm, there are mainly two research directions, single mode and multimode. Classic pattern matching algorithms contain the KMP algorithm, BM algorithm, AC algorithm, and so on.

The KMP algorithm [23] is proposed by Knuth et al.. The algorithm is based on prefix matching. The KMP algorithm matching principle is an approach run within the matching window from the testing text left to right to match. Inspired by KMP algorithm, Boyer and Moore [24] proposed a new fast string matching algorithm: the BM algorithm. The BM algorithm is a suffix-based matching algorithm, which considers the position of the characters that may appear in the string pattern and can glide the body dramatically.

The main idea of the BM algorithm is to conduct the matching comparison from the left position *i* in the text data with patterns from the right to left. At the beginning, the pattern is aligned with the text in the leftmost side. When mismatching occurs, the next match position should be *i* + dist⁡[*ρ*_*i*_] in the text, which is equivalent to right sliding of the pattern by a distance of dist⁡[*ρ*_*i*_] and a skip of dist⁡[*ρ*_*i*_] characters without comparison. If the character does not appear in the pattern, or appears at the end of the pattern, then the slide to the right would be with the greatest distance of *m*.

The Aho-Corasick (AC) algorithm [25] can be regarded as an extension of the KMP algorithm for the problem of multipattern matching. The AC algorithm uses a TRIE data structure to organize all the pattern strings. It reduces the cycle the strings require in all modes within each matching window match within the window of the TRIE data structure. The AC algorithm constructs a dendriform finite state machine, of which the different internal nodes are interconnected. These connections enable the failed match to quickly jump between the other branches of the tree which share a common prefix. Therefore this also means that the automata do not need to backtrack during the transition of pattern matching.

## 3. System Architecture and Detection Algorithm Process

**Figure 2:**
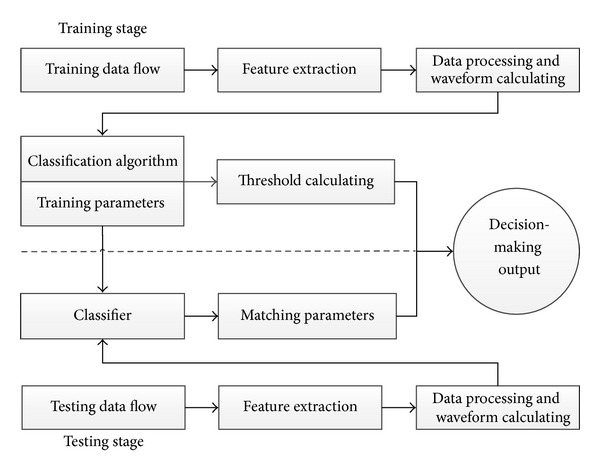
System framework.

This paper proposes a signal waveform packet classification algorithm and on this basis designs a real-time network packet detection system. The necessary raw data can be obtained by real-time capturing network packets and then the final purpose of matching and classification accomplished after system processing and computation. Through proper training the system can detect the behaviour of the network applications of users. The design of the framework of this packet inspection system is shown in Figure 2.

As with the conventional detection system, the system can be divided into two parts: training part and testing part.

The training part is used to establish the classification model of the system for detection. After data processing, the statistical waveform features will be extracted and classifier rules will be established, and then the decision threshold should be designed.

The testing part will monitor the real-time data traffic and extract feature information of the message, which will be fed to the classifier for matching. According to the matching extent of the testing data and the default data of the classifier, as well as the training threshold value, the system will conduct the final decision making, to achieve the purpose of monitoring the network behaviour.

The system implemented by this paper is constructed in an environment established by ourselves. The procedure of the acquisition and analysis of the data at the gateway can be divided into five steps.

1. Capture data packets. Use* libpcap* to capture network packets on a Linux platform, and record the content of the data packets.
2. Feature extraction and preprocessing. Extract the feature information of the system from the network packet header which is required for the matching judgment, and then conduct operation and quantization processing according to the different characteristics and obtain the required feature values of this issue.
3. Calculation of data statistics. After obtaining a certain amount of network traffic data frames, record the waveform of the feature information. Then calculate the appropriate amount of statistical data according to the digital characteristics of the feature information, which will be used as input to the classifier.
4. Classifier determination. Formulate classification strategies according to the various features and statistics collected, and design different classifiers for the matching of various inputs. Decision-making is to determine the testing data by training and experimentation, so that the analysis of network packets can be implemented.
5. Results and output. Generate users' network behaviour logs, summarize the behaviour of the gateway network application, and then output the results.

Through the several steps above, the system can implement the real-time monitoring of network traffic flowing through the established environment and enable the identification of the user network application behaviour more effectively.

**Figure 3:**
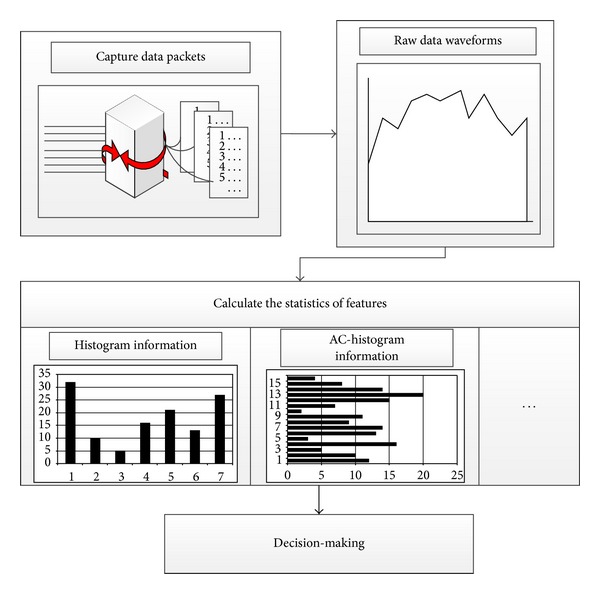
Implementation of matching algorithm.

The core detection contents of the system are the classification and matching of packet patterns. The specific process of the packet classification algorithms designed in this paper and related algorithms are shown in Figure 3.

From the beginning of the collection of the data to the final output of the decision-making, in this paper, a variety of algorithms are implemented for data conversion, calculation, and matching. The following section will give a detailed description and analysis of the algorithms from several aspects, such as the original data processing and the process of pattern matching.

## 4. Proposed Algorithm

### 4.1. Original AC Algorithm

In the analysis and matching of specific network behaviour, AC automaton is utilized. The algorithm was developed in Bell Labs in 1975 and is one of the most famous multimode matching algorithms.

The basic idea of the algorithm is as follows. In the preprocessing stage, the AC automaton algorithm will establish three functions: a steering function which is defined as *goto*, a failure function named *fail*, and an output function *output*, which constructs a tree-type finite automaton. In the searching stage, it firstly locates all the occurrence positions of the keyword in the text by scanning the text through an intersecting utilization of the three functions.

This algorithm has two features: one is that during the process of scanning the text completely it does not need to backtrack, and the other is that the time complexity will be *O*(*n*), and the time complexity has nothing to do with the number or length of the keyword.

The implementation of the AC multipattern matching algorithm can be divided into two stages: preprocessing and matching. In the preprocessing stage, a finite state machine is generated according to the string pattern to be matched; in the matching stage the state machine will conduct a state transition according to the input text string. When a certain state is reached, if the status has a matching pattern string, then the match succeeds. The AC state machine includes the three functions of *goto*, *fail*, and *output*.

(*A*) *goto* *Function*. The AC-matching algorithm, which takes character as its processing unit, hypothesizes that the string group *P* contains *n* patterns, and state machine will be generated by the use of pattern string groups consisting of five steps (shown below).

1. If *n* ≤ 0, return error; *i* = 0;
2. if *i* ≤ 0, take out the string pattern *P*_*i*_ and set the current state *S* = 0; otherwise, end the generation stage;
3. take the next character *C* of the *P*_*i*_, if *C* exists, then *S* = *goto*(*S*, *C*); otherwise, jump to step (2);
4. if *S*! = −1 (−1 denote null state), jump to step (3);
5. *goto*(*S*, *C*) = *NewState* (NewState: generate a new state and jump to step (3)).

(*B*) *fail* *Function*

1. Define the shortest length of the path from state 0 to the state *S* as the depth of state *S*;
2. set all states whose depth is 1 to *fail*(*s*) = 0; calculate the value of fail from low to high according to depth;
3. the fail value of the depth *d* state is calculated by state *r* whose depth is *d* − 1;
4. set *state* = *fail*(*r*, *a*);
5. execute *state* = *fail*(*state*) zero to several times until *goto*(*state*, *a*)! = *Fail*;
6. set *fail*(*s*) = *goto*(*state*, *a*).

(*C*) *output* *Function*

1. During the calculation of the *goto* function, after the completion of a mode string added into the state tree, add the current mode string as the output for the last state of that mode string;
2. during the calculation of the *fail* function, when the calculated *r* equals *fail*(*s*), add the output mode string of *r* to the state of *s*.

### 4.2. Proposed Basic Approach: AC Waveform

The flow of the AC waveform method is as follows.

The system processing of the AC Waveform is described by the following notations: App^(*i*)^ denotes the *i*th application and *S*_*T*_^(*i*)^ is *i*th training stream, label *T* means the stream is for training, and App_*S*_*T*__^(*i*)^ is used to represent the true application of the training stream, where *i* = 1,2, 3,… is the serial number of the training applications. Thus, consider (*S*_*T*_^(*i*)^, App_*S*_*T*__^(*i*)^) as a training sample labeled by App_*S*_*T*__^(*i*)^, which will be the input of the system in the training stage. (Note: in the following description, without special instruction, the symbol ∶ = means the assignment operation and the notation = represents truth assertion, that is, the comparison operation between the left and right sides of the value, and *Value*{*condition*(*s*)} means that the value can only be obtained provided the condition(s) inside the brackets is(are) met.)

Training stage. First, a preprocessing procedure is conducted on the data stream which will be denoted by the following equation:

(1) $$\begin{array}{c} \left(L_{T}^{\left(i\right)},{\text{App}}_{S_{T}}^{\left(i\right)}\right)∶=\Gamma_{e}\left(S_{T}^{\left(i\right)},{\text{App}}_{S_{T}}^{\left(i\right)}\right), \end{array}$$

where  *L*_*T*_^(*i*)^ represents the extracted frame length information.

The next step will be the waveform generation process, which can be demonstrated by the following equation:

(2) $$\begin{array}{c} \omega_{T}^{\left(i\right)}:=\Lambda_{\omega }\left(L_{T}^{\left(i\right)},{\text{App}}_{S_{T}}^{\left(i\right)}\right). \end{array}$$

Consider *ω*_*T*_^(*i*)^ as the generated waveform by conducting the process of Λ_*ω*_ function with the frame length information *L*_*T*_^(*i*)^ extracted in the last step. Then it converts the waveform information into short patterns, and we use the Δ_*c*_ function to represent this process; the string segmentation strategy involved will be described in detail in the following sections. Here, the following equation is simply used to illustrate this procedure, regardless of the implementation details of the process:

(3) $$\begin{array}{c} {\delta \left[P_{m}\right]}_{T}^{\left(i\right)}∶=\Delta_{c}\left(\omega_{T}^{\left(i\right)}\right), \end{array}$$

where *δ*[*P*_*m*_]_*T*_^(*i*)^ denotes a string array of a group of short patterns with label App_*S*_*T*__^(*i*)^ generated in the Δ_*c*_ process and *P*_*m*_ is the *m*th short pattern with *m* = 1,2, 3,….

Then, we feed *δ*[*P*_*m*_]_*T*_^(*i*)^ into the system to establish AC automaton *ϕ*_*T*_^(*i*)^, which can be expressed by

(4) $$\begin{array}{c} \phi_{T}^{\left(i\right)}∶=\Phi_{e}\left({\delta \left[P_{m}\right]}_{T}^{\left(i\right)}\right). \end{array}$$

Similarly, in the test stage, *S*_*D*_^(*j*)^ is used to denote the testing data stream, where the subscript of label *D* signifies that the stream is for detection. Then, the testing data stream will be fed into the testing system. The same data preprocessing steps will be conducted, including frame length information extraction and waveform establishment (see (1) and (2)); we will conduct a data conversion procedure, which will generate the short testing patterns. Similarly, the process can be denoted by the following equation:

(5) $$\begin{array}{c} {\delta \left[P_{n}\right]}_{D}^{\left(j\right)}:=\Delta_{c}\left(\omega_{D}^{\left(j\right)}\right). \end{array}$$

Then, the data is input into the established AC automaton in the training stage, and the total number of matches will be counted:

(6) $$\begin{array}{c} C_{S_{D}^{\left(j\right)}}^{\left(i\right)}∶=\underset{n}{\sum }1\left\{M_{c}\left({\delta \left[P_{n}\right]}_{D}^{\left(j\right)},\phi_{T}^{\left(i\right)}\right)\right\}, \end{array}$$

where the *Matching* function refers to the matching process after the data is fed to the AC automaton. *MR*_(*j*)_^(*i*)^  is used to represent the decision-making result by comparing the counted result with the set threshold, which can be denoted by

(7) M(j)(i)∶={1,CSD(j)(i)>threshold0,CSD(j)(i)≤threshold(i=1,2,3,…;j=1,2,3,…),

where the value of {1,0} refers to a testing data stream *S*_*D*_^(*j*)^ match or mismatch with App^(*i*)^ according to its following condition holds.

Then, it uses the following equation:

(8) $$\begin{array}{c} R_{S_{D}^{\left(j\right)}}≔{\text{App}}^{\left(i\right)}\left\{1=M_{\left(j\right)}^{\left(i\right)}\right\} \end{array}$$

to represent the output of the testing result, which means that the testing stream *S*_*D*_^(*j*)^ belongs to application App^(*i*)^, labelled by *i* when the value of decision-making result *M*_(*j*)_^(*i*)^ equals 1.

**Figure 4:**
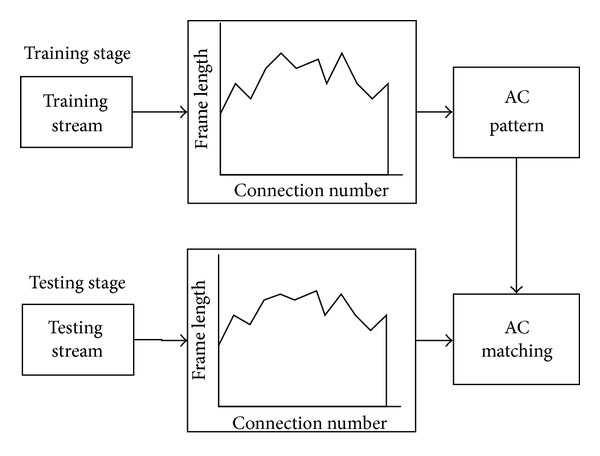
AC waveform flow.

In short, after the standard file is loaded, the system extracts the length information of the data frame and converts it to a string, with a certain number of characters as the unit record and short waveform, and counts the number of occurrences of the short waveform (back-off packet length every time). So the standard waveform and its number of occurrences would be obtained through the training and AC automata established on this basis. In the testing stage, the system conducts string conversion with other data and carries out the AC match, as well as recording pattern matching occurrence. The matching number and the standard number are calculated, in order to obtain the square of the difference, which will be considered as the basis of the judgment. Both the differences between similar applications and different applications will be recorded. Likewise, the best judgment threshold value will be found to distinguish the different types of application through repeated experiments. On one side of the threshold value, they are similar applications, and on the other side they do not fall into this category (see Figure 4).

### 4.3. Advanced Approach: AC-Histogram Waveform

As with the description of the AC waveform, similarly, Symbol App^(*i*)^ is also used to represent applications and *S*_*T*_^(*i*)^ is the  *i*th  training stream, whereby label *T* means that the stream is for training and App_*S*_*T*__^(*i*)^ is used to represent the application of the training stream with *i* = 1,2, 3,… which is the serial number of the training application. Thus, (*S*_*T*_^(*i*)^, App_*S*_*T*__^(*i*)^) is regarded as a training sample labelled by symbol App_*S*_*T*__^(*i*)^, which will be the input of the system in the training stage.

In the training stage, after the same data preprocessing, which includes frame length information extraction, as well as waveform establishment (see (1) and (2)), however, we will conduct a different AC automaton establishment process, the Ψ_*e*_( ) function, and the new automaton will be denoted by *ψ*_*T*_^(*i*)^ in the following equation:

(9) $$\begin{array}{c} \psi_{T}^{\left(i\right)}≔\Psi_{e}\left({\delta \left[P_{m}\right]}_{T}^{\left(i\right)}\right),\\ \text{for}\;\;\text{each}\;\;P_{m}:{C\left[P_{m}\right]}_{T}^{\left(i\right)}∶=\;\underset{m}{\sum }1\left\{O_{c}\left({\delta \left[P_{m}\right]}_{T}^{\left(i\right)}\right)\right\}, \end{array}$$

during which *O*_*c*_ function is added to counting the number of occurrences of each patterns, and an array symbol *C*[*P*_*m*_]_*T*_^(*i*)^ is used to store the statistical results for each pattern, which will provide the basis of the data for the establishment of the histogram in the following step.

The next step will be the histogram establishment process, which can be illustrated by

(10) $$\begin{array}{c} H_{T}^{\left(i\right)}∶=H_{e}\left({C\left[P_{m}\right]}_{T}^{\left(i\right)},{\delta \left[P_{m}\right]}_{T}^{\left(i\right)}\right). \end{array}$$

In the test stage, *S*_*D*_^(*j*)^ is used to represent data stream for detection and feed it into the test system. Through the same data preprocessing, which includes frame length information extraction, as well as waveform establishment (see (1) and (2)), the quantized data of short patterns can be obtained by carrying out data conversion on the *ω*_*D*_^(*j*)^ established:

(11) $$\begin{array}{c} {\delta \left[P_{n}\right]}_{D}^{\left(j\right)}∶=\Delta_{e}\left(\omega_{D}^{\left(j\right)}\right). \end{array}$$

Then, input *δ*[*P*_*n*_]_*D*_^(*j*)^ into each of the AC-histogram automata, for which the symbol is *ψ*_*T*_^(*i*)^, constructed in the training stage, and simultaneously count the number of matches for each short pattern, which can be illustrated by the following equation:

(12) $$\begin{array}{c} \text{for}\;\;\text{each}\;\;P_{n}:{C\left[P_{n}\right]}_{S_{D}^{\left(j\right)}}^{\left(i\right)}∶=\underset{n}{\sum }1\left\{M_{c}\left(\delta {\left[P_{n}\right]}_{D}^{\left(j\right)},\psi_{T}^{\left(i\right)}\right)\right\}, \end{array}$$

where the *M*_*c*_ function refers to the matching process after the testing short patterns for testing is fed into AC-histogram automaton.

Then, a histogram of the counted results  *C*[*P*_*n*_]_*S*_*D*_^(*j*)^_^(*i*)^ is established by

(13) $$\begin{array}{c} H_{D}^{\left(j\right)}∶=H_{e}\left({C\left[P_{n}\right]}_{S_{D}^{\left(j\right)}}^{\left(i\right)},{\delta \left[P_{n}\right]}_{D}^{\left(j\right)}\right). \end{array}$$

After obtaining *H*_*D*_^(*j*)^, the Euclidean distance between the *H*_*T*_^(*i*)^ constructed in training stage and *H*_*D*_^(*j*)^ will be calculated and the process can be denoted by the *d* function. Then, we also use *M*_(*j*)_^(*i*)^ to represent the decision-making result by comparing the computation result with the set threshold, which can be denoted by

(14) M(j)(i)∶={1,d(HD(j),HT(i))>threshold0,d(HD(j),HT(i))≤threshold(i=1,2,3,…;j=1,2,3,…).

Finally, use the following equation:

(15) $$\begin{array}{c} R_{S_{D}^{\left(j\right)}}∶={\text{App}}^{\left(i\right)}\left\{1=M_{\left(j\right)}^{\left(i\right)}\right\} \end{array}$$

to represent the output of the testing result, which means that testing stream *S*_*D*_^(*j*)^ belongs to application App^(*i*)^, labelled by *i* when the value of the decision-making result *M*_(*j*)_^(*i*)^ equals 1.

**Figure 5:**
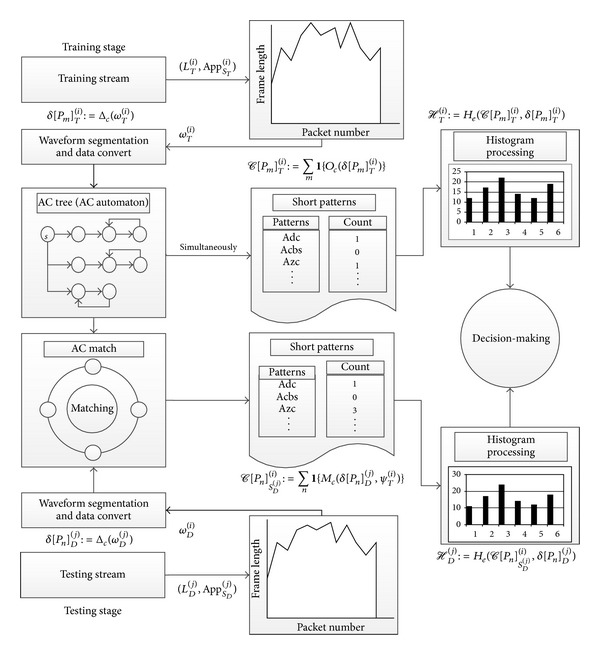
AC waveform histogram flow.

In brief, after processing the packet frame length information, establish the automaton according to the training set. In the testing stage, feed the data into the automaton for matching to achieve classification and the determination of decision. This proposed algorithm accomplishes fuzzy matching by dividing string into shorter substrings and counting the matching extent of the substrings. The specific implementation of the algorithm is shown in Figure 5.

The algorithm execution process is also divided into two parts, training and testing. The training part mainly includes the following steps: the conversion of waveform information, partitioning of the pattern string, substring processing, establishing the AC-histogram automaton, and recording the original AC-histogram statistics.

The detection stage (testing stage) includes several steps: conversion of waveform information, AC automaton matching, and record matching of histogram statistics. In order to achieve the purpose of the final classification, the system will match the histogram statistics of the waveform with the original mode AC-histogram statistics of the waveform and make a comparison to measure the extent of the subsequent matching. The process will be specifically performed as in Figure 5.

### 4.4. Example for Data Preprocessing and Short Pattern Conversion

The data distribution of the frame length is wide range, which approaches continuous numeric values, and this feature can describe much of the information about the data packet; thus, we can take it as a key part in the detection process. After the mathematical processing described in the previous section, the system will effectively quantify the data again and reduce the range of the data to 1–35, which it subjects to the reservation about most of the information of features. Due to the continuous characteristics of the values, as well as for keeping the chronological correlation of the network traffic, the system uses multimode matching algorithm for data classification [26].

In the preprocessing stage, after the system reads the raw standard data, firstly, the waveform of the frame length is quantified, and for ease of matching the system converts the data from a decimal number into a character, which means that 1 to 35 can be mapped to a–z and A–I, a total of 35 characters. Specifically, according to the data distribution used to conduct the conversion, the conversion rules are as follows.

51 bytes–200 bytes (of which the interval is 10 bytes) are converted to “a” to “o”; that is, if the frame length of the data is between 51 bytes and 60 bytes, the frame length information will be recorded as the letter “a,” 61 bytes to 70 bytes will be denoted by b, and so on; 201 bytes–2000 bytes (of which the interval is 100 bytes) are converted to “p” to “z” and “A” to “G”; that is to say, if the frame length of the data is between 201 bytes and 300 bytes, the frame length information will be recorded as “A,” the 301 bytes to 400 bytes will be denoted by “B,” and so on.

Those less than or equal to 50 bytes will be denoted “H”; and those greater than 2000 bytes will be recorded as “I.”

So the data frame length information will be effectively quantified, and due to the presence of a divided interval, the fuzzy matching can be effectively conducted in order to reduce interference to the system caused by the noisy frame data.

**Figure 6:**
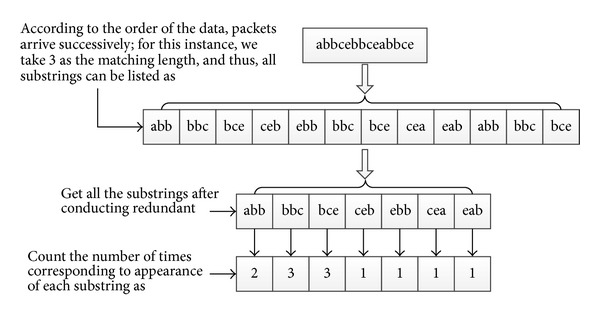
Example of quantization process of frame length information.

Thus, we can obtain the string of the flow of the frame length. For this example, see Figure 6 for more details.

Thus, in this procedure of data processing, the algorithm firstly converts the integer of the length information of the data frame to a string, then takes a certain number of characters as the unit of the recording pattern, and counts the pattern mode and its number of occurrences. Then, it regards it as the standard and uses these patterns to establish the AC automata. At the testing stage, it conducts string conversion with other data, uses the AC algorithms to count matching number of each pattern, and then counts the histogram of the pattern's number of occurrences in chronological order. Furthermore, the algorithm calculates the sum of the square of the difference of the remaining label histogram and according to this makes the decision. Thus, the best judgment threshold value is found through repeated experiments.

### 4.5. Selection Strategy of the Decision Threshold

The algorithm selects the optimal threshold of the experiments for judgment through an exhaustive method, which is described as follows.

Suppose there are two types of data named as A and B, and each one of them is one-dimensional. The range of data of class A is A_max⁡_ ~ A_min⁡_, while the range of the class B data is B_max⁡_ ~ B_min⁡_.

If there is no crossover between the two types of data, we may wish to assume that A_max⁡_ is less than B_min⁡_, then taking a certain value between them as the judgmental threshold, and the accuracy of distinguishing it is 100% otherwise there should be two cases B_max⁡_ ≥ A_max⁡_ ≥ B_min⁡_ or B_max⁡_ ≥ A_min⁡_ ≥ B_min⁡_. We will enumerate all the values between A_max⁡_ and B_min⁡_ or A_min⁡_ and B_max⁡_, which will be regarded as the judgmental threshold sequentially in following steps, and then record the number of samples with an error, noting the smallest number which is favoured as the threshold.

### 4.6. Correctness of Proposed Algorithm

In this section, we prove the correctness of our proposed algorithms, which include AC waveform algorithm and AC-histogram algorithm.

#### 4.6.1. Proof of Correctness of AC Waveform Algorithm

The changes of the data frame length information *L*_*T*_^(*i*)^ within a period of detection will be regarded as the determination basis for the network behaviour of an application. After the quantization process, the Γ_*e*_ function, the waveform information *ω*_*T*_^(*i*)^ of the data frame length will be obtained, that is, the behavioural statistics information. Then, data conversion (Δ_*c*_ function) is conducted for the waveform to obtain the behavioural string data. Through fixed-length segmentation, the substring patterns of behaviour *δ*[*P*_*m*_]_*T*_^(*i*)^ will be obtained. On this basis, establish the AC automaton *ϕ*_*T*_^(*i*)^ by the utilization of the redundant pattern strings. In the testing stage, by carrying on the same data preprocessing for detecting data stream, the pattern strings of the testing data  *δ*[*P*_*n*_]_*D*_^(*j*)^ will be obtained. Then, input the patterns into the established AC automaton *ϕ*_*T*_^(*i*)^ to conduct the pattern match, and count the number of matching occurrences *C*_*S*_*D*_^(*j*)^_^(*i*)^∶ = ∑_*n*_1{*M*_*c*_(*δ*[*P*_*n*_]_*D*_^(*j*)^, *ϕ*_*T*_^(*i*)^)}. When the number of matching is larger than a certain threshold, we have reason to believe that the behaviour of the test data waveform has reached a certain level of matching with the waveform of the training samples, on the basis of which the AC automaton is established. And the degree of matching allows us to accept the assumption that the detected data belong to a certain application.

To a certain extent, the AC waveform detection method observes more of the overall trends in behavioural waveforms of the data frame length information within a certain period of time, rather than focusing on the detection of the detailed information of matching within certain short time intervals.

#### 4.6.2. Proof of Correctness of AC Waveform Histogram Algorithm

The data preprocessing of AC waveform and histogram method is the same as the AC waveform method described above. Similarly, it treats the changes in the data frame length information *L*_*T*_^(*i*)^ within a period of detection as the basis of determination for the network behaviour of an application. After the process of quantization Γ_*e*_, the waveform information on the data frame length, that is, the behavioural statistics information *ω*_*T*_^(*i*)^, can be obtained. Then, data conversion (Δ_*c*_ function) for the waveform is conducted to obtain the behavioural string data. Through fixed-length segmentation, the pattern substrings of behaviour *δ*[*P*_*m*_]_*T*_^(*i*)^ will be obtained. On this basis, the establishment of the AC-histogram automaton *ψ*_*T*_^(*i*)^ will be conducted by the utilization of the redundancy pattern strings. However, the difference is that during the process of establishment of AC-histogram automaton Ψ_*e*_( ), more detailed information is simultaneously recorded, that is, the number of occurrences of each string pattern *C*[*P*_*m*_]_*T*_^(*i*)^∶ = ∑_*m*_1{*O*_*c*_(*δ*[*P*_*m*_]_*T*_^(*i*)^)}, and establish histogram (*H*_*e*_ function) for them, which will be the basis of decision-making for matching. In the testing stage, we will carry out the same preprocessing as for the detecting data stream, and the pattern strings *δ*[*P*_*n*_]_*D*_^(*j*)^ of the testing data will be obtained. Then, the patterns are input into the established AC-histogram automaton *ψ*_*T*_^(*i*)^ to conduct pattern matching, similar to the training stage, the number of matching occurrences for each pattern is counted, *C*[*P*_*n*_]_*S*_*D*_^(*j*)^_^(*i*)^∶ = ∑_*n*_1{*M*_*c*_(*δ*[*P*_*n*_]_*D*_^(*j*)^, *ψ*_*T*_^(*i*)^)}, and a histogram is constructed. Then, it makes comparison between the histograms built in the two stages, and the Euclidean distance is taken as the measure of the degree of matching. When the computed value of the distance is less than a certain threshold, it thus has reason to believe that the behaviour of the test data waveform achieves a certain level of match with the waveform of the training samples based on which AC-histogram automaton is established. And the degree of matching allows us to accept the assumption that the detected data belongs to the certain application.

In contrast, to a certain extent the AC waveform histogram method requires more demanding conditions for matching determination. Not only the overall trend of behavior waveform within a certain period of time reaching a certain matching degree is required, but also the condition that small zones of the behaviour waveform need to achieve a certain level of matching. Depending on these conditions, the conclusion of decision-making for determination of matching can be drawn. That is to say, we conduct the measure of matching in smaller intervals, in order to achieve a higher accuracy.

## 5. Advanced Analysis

### 5.1. Extraction and Processing of the Packet Feature Information

#### 5.1.1. Original Packet

**Figure 7:**
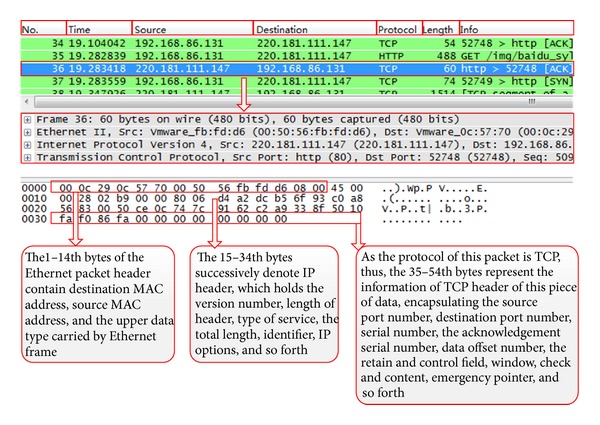
Parse packets result from Wireshark.

The original data can be obtained by capturing packets at the gateway. For ease of interpretation, we use* Wireshark*, which is network software used for packet analysis, shown in Figure 7.

The hexadecimal data behind the comprehensive information is the contents of the data frame, such as data packet number, frame length, and arrival time.

Feature information needed by this system will be extracted and calculated from data packet similar to the above, and the following sections will provide specific description.

#### 5.1.2. Feature Extraction and Quantification

Feature extraction is an important part of the input for the classifier, which has a direct impact on the quality of the final determination. This paper referenced some of the methods, Cannady selected nine data features; not only because they are normally present in the network packets but also because they provide a complete description of the packet transmission information [27], which includes protocol type, source port, destination port, source IP address, destination IP address, ICMP type, ICMP code, the length of the original data, and the data portion [28]. Today, however, due to the widely used characteristics of distributed technology, features such as source and destination IP address become nonrepresentative. In addition, KDD-CUP-99 processed the data of the 1998 DARFA intrusion detection evaluation program and selected 41 dimensional feature data with which we conduct detection analysis [29]. However, due to the cumbersome processing of the experimental data and offline detection, it is usually too much for academic research. According to the topic of statistical-based information and the real-time requirements, this paper extracted 9 data features from the data packets as the object for study. These features are a good representative of the information contained in the data transmissions, and the specific features are listed as in Table 1.

In order to facilitate the subsequent operation of the algorithm, the system will carry out the decimal conversion processing for each feature, wherein the hexadecimal data of the Ethernet protocol type is converted into decimal numbers; that is, IP = 2048, ARP = 2054, and RARP = 32821. In addition, the binary data of the TCP control field is converted into decimal numbers. The remaining feature information is denoted directly by its original decimal value. Through the above quantization method, the system can obtain 9-dimensional decimal data from each data frame based on the selected features, and the statistical waveform of the various characteristics of the segment data packets can be obtained after a certain flow of data packets.

### 5.2. Packets Matching Algorithm

After obtaining waveform information for the original features of the network segments, the core part of the system will be conducted, the pattern matching calculation section. To this end, the different computation and statistical programs were designed for various features of the original waveform in order to obtain the statistical feature values which carry significant characteristics of the segment of the data flow. Then, these data are used to set the classification rules of the classifier. Through a great deal of training and experimentations, the means of the measure of the matching degree are set in addition to the threshold determined, as well as the implementation of network packet matching and outputting of the results.

With regard to statistical feature extraction, the system adopted the histogram method to determine part of the information. However, for the data frame length information, this study designed a unique method, that is, the AC match-histogram method, and utilized the above program to complete the calculation process of the feature statistics. In addition, multiple strategies for measuring the degree of matching have been designed, such as the direct calculation of the Euclidean distance between the statistical waveforms. See below for a detailed description of the algorithm.

#### 5.2.1. Design and Calculation of the Feature Information of the Statistical Waveform

After a certain time span of the data packets is obtained, the system will extract and quantify the feature information of each data packet, so that the statistical waveform information of each feature in the network traffic segments will be obtained. According to the distribution of the facts and characteristics of the figures for each feature, the system classified the features into three types, in order to obtain different statistics.

(1) Features of Data Protocol Type. The system will conduct a statistical investigation and analysis of the Ethernet protocol type, the IP protocol type, and ARP/RARP opcode of the data packet. Since the data of these features is discrete, which also keep the mutually exclusive relationship, the data thus can be divided into different 19 categories. To this end, the system regards IP protocol type, ARP/RARP operation as determination conditions, to classify and analyze the data packets.

When each packet is obtained, the system will carry out the first division according to its Ethernet type, and thus the data packet will be classified into three branches, which include IP packets (Ethernet type is 2048), ARP packets (Ethernet type: 2054), and RARP packets (Ethernet type 32821), and does not count the rest. For the first branch of the IP packet node, according to its specific IP protocol, the data is once again divided into TCP packets (IP protocol type 6), UDP data packet (IP protocol type 17), and ICMP data packets (IP protocol 1), and the rest will not be counted. For the second branch of the ARP packet node, according to its specific protocol, the data packet can be classified into specific types with regard to the operation requested: operation package (opcode = 1) and response operate packet (opcode = 2). Similarly, the third branch of the RARP data packet can also be divided into the requested operation packet (operation code is 3) and response operation packet (operation code is 4).

**Figure 8:**
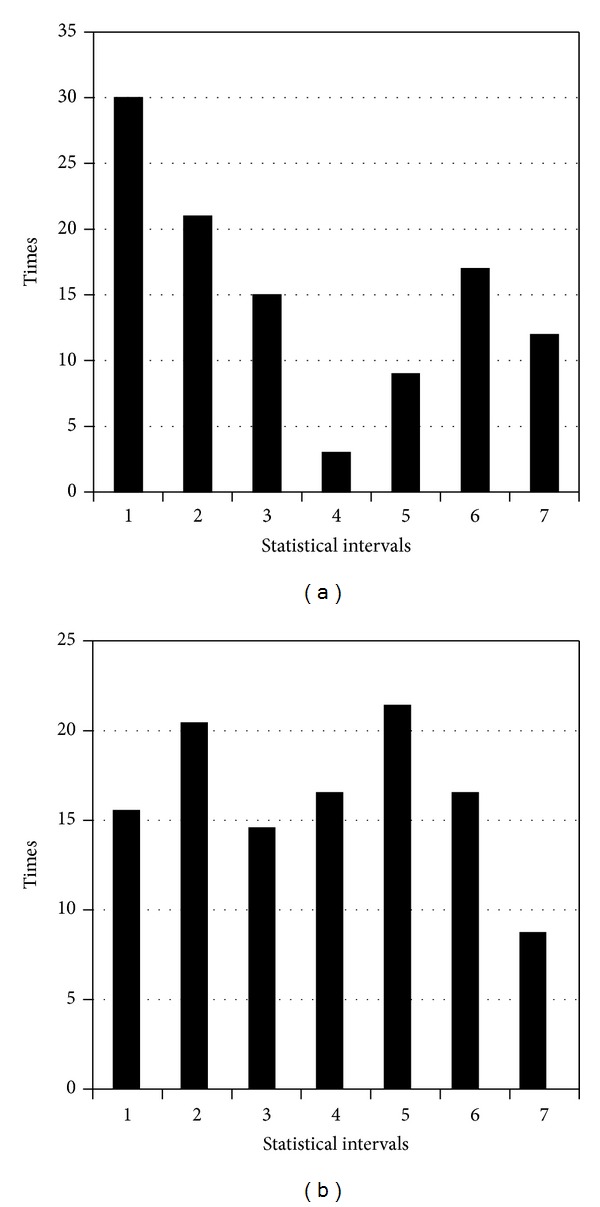
(a) Protocol type feature: port number feature. (b) Statistical waveform.

Thus the system will use the several data features mentioned above and the designed classification rules to ultimately summarize and allocate each data packet into one of seven categories. However, when the original packets of data traffic set by the system are obtained, the system will determine the number of data frames classified into each category for each segment, and after organization a piece of statistical information with a length of 7 can be obtained. See Figure 8(a) for details of the histogram diagram.

(2) Port Number and TCP, ICMP Specific Features of Information, including the Message Source and Destination TCP/UDP Port Number, TCP Control Field, ICMP Code, and ICMP Type. This part can extract seven sets of data from the packets, and all of them are discrete. The respective description of each of feature is listed as follows.

- TCP/UDP source and destination port number ranges from 0 to 65535, according to the type and distribution of the port. The port number can be divided into seven intervals. After the equalization process, the interval ranges are 0–21, 22–50, 51–100, 101–500, 501–1023, 1024–49151, and 49152–65535. Thus the system can obtain histogram statistics of the data port information of this data flow, as shown in the diagram in Figure 8(b).
- ICMP type features, divided according to the value of the data distribution, by equalizing the height, and the intervals are divided as follows: 0–7, 8, 9–18, and 19–255, that is, four intervals.
- ICMP code, which is very simple and does not require much processing, could be classified into five categories, which can be denoted by five intervals including 0, 1, 2, 3, and >3.
- TCP control field. According to the different senses of the 6-bit flag segment characters conduct statistical computation according to whether each bit is effective, which is divided into six intervals, URG = 1, ACK = 1, PSH = 1, RST = 1, SYN = 1, and FIN = 1, so that the system can obtain three statistical histogram waveforms similar to the port number of the data packets and ICMP and TCP information features.

(3) Frame Length Information. Frame length information in bytes, its value on the basis of the experiments in this paper, is almost continuous, ranging from 50 to 2000 bytes. Similarly, according to a large number of experimental data, the optimal division of intervals is as in Table 2.

Thus the length information of each frame can be reduced to a range of 35 discrete data.

### 5.3. System Output

With the various statistical features of information matched, the system will obtain the corresponding result of the matching. Through the generalization of these results, the final decision about whether the data packet matches the corresponding behaviour will be made. The consolidated results of the matches of various statistical features can be denoted by the following equation:

(16) $$\begin{array}{c} \mu =\mu_{1}*w_{1}+\mu_{2}*w_{2}+\cdots +\mu_{n}*w_{n}=\overset{n}{\underset{i=1}{\sum }}\mu_{i}*w_{i}, \end{array}$$

where *μ*_*i*_ is the match situation of each of the statistical features; if there is a matching case, its value will be 1; otherwise, it will be 0.  *w*_*i*_ is the weight of each item; in this study, the features are similar; that is, no major core features exist, so the values will be set as

(17) $$\begin{array}{c} w_{i}=\frac{1}{n}=\frac{1}{9} \end{array}$$

which means that the weights of the features are equal to each other. This trial extracted 9 features of information of the statistical waveform, therefore, basically set each of the weights to 1/9.

After the training process we can determine the threshold of *μ*, which is the consolidated matching value, and make the final decision with regard to the match results, so that we can accomplish the objective of the detection of network data packets.

## 6. Evaluation

According to the description of the system framework and the detection processes in the previous sections, in this study, we built a corresponding network environment and implemented the network packet detection system on the gateway. The system utilizes related algorithms to conduct data preprocessing, feature statistics calculations, matching determination, and the output of the final test results. We also illustrate the performance of the algorithms by a comparison experiment with an approximation algorithm. We built the network environment in our lab and implemented a real-time packet inspection system based on the Linux operating system on the gateway and used PHP to build the front end operation webpage of the system in order to make the interface user-friendly.

### 6.1. Environment Setup

**Figure 9:**
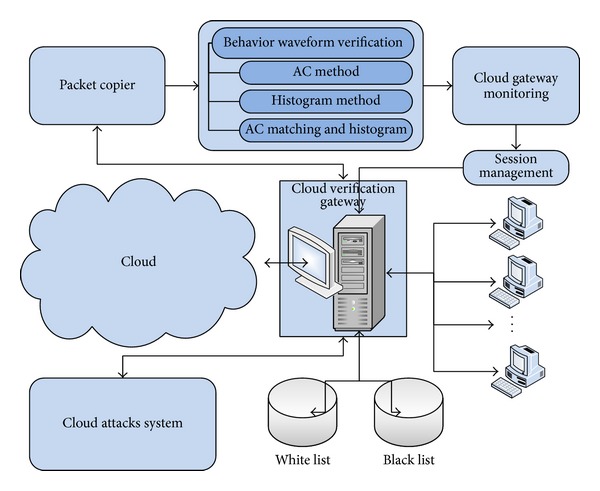
Diagram of evaluation environment.

Experiments were carried out in an environment containing a PC, which will be the gateway through which other PCs can connect with the external network and be used. See Figure 9 for details of the environment setup.

The gateway PC uses double network cards to implement subnet data transmission. The intrusion detection system designed in this paper is also set up on this PC.

### 6.2. Packet Capture

The system utilizes* libpcap* to obtain the data packets.* Libpcap* is a network packet capture and filtering package which was originally developed by a research team at the Lawrence Berkeley Lab.

**Figure 10:**
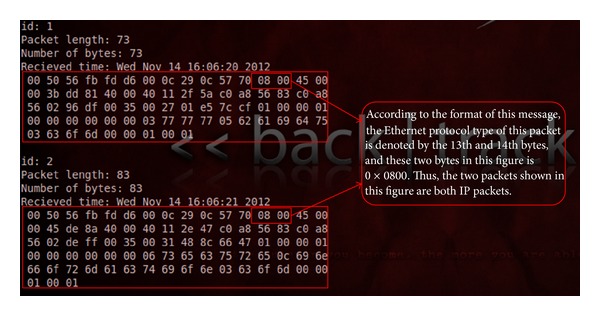
Data packet information.

This system will mainly use the* pcap_findalldevs* function to search the network devices and the* pcap_loop* function to perform packet capture. The data packets flowing through the network card will be printed out, as shown in Figure 10.

The packet number, length, and capture time information will be first and then the packet specific hexadecimal content. The feature information required by the system on the packets will be read out and calculated from the data packet content. We can extract the various characteristics required by the algorithm which enables information of the packet to be described.

### 6.3. Data Preprocessing

According to the features extraction algorithm designed in the previous section, we will quantify 9-dimensional values of the data packets features, which will be converted into decimal integers after processing (see Table 3 for detailed information).

We can thus obtain data on the characteristics of the packet in a segment of network traffic, and the system will record it as shown in Table 4.

According to the feature value information of each data frame in the traffic, we can obtain the value of the various features of the characteristics of the waveform in the flow, thereby performing the computation of the amount of feature statistics.

### 6.4. Calculation of Packet Features

The system uses the features statistics algorithm designed in the previous section in order to calculate the statistical information of each of the values of features needed to be classified. For different features, different statistics calculations are applied. The conversion outcomes of the frame length information of the data flow generated by the log MSN application are following, and the system will consider 50 data frames as a process unit for evaluation. String patterns generated by the frame length information are listed as follows (length 3); see Table 5.

**Figure 11:**
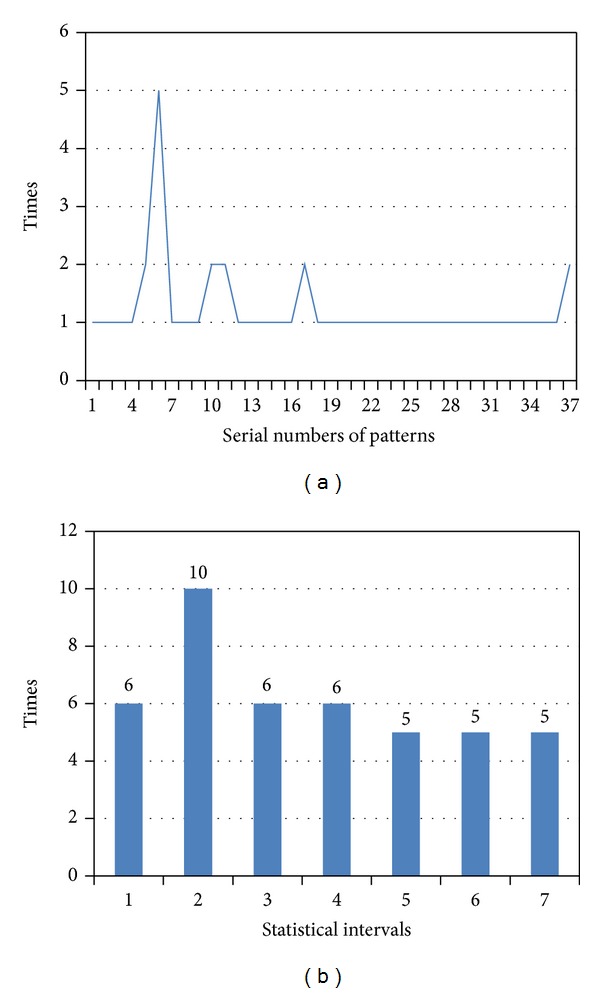
(a) String pattern occurrences waveform; (b) AC matching-histogram information of frame length.

The numbers of occurrences of each pattern are shown as waveforms in Figure 11(a).

As shown in the above waveform, according to the chronological order of the first occurrence of the string pattern, a uniform interval histogram is constructed (interval length is 5); that is, Figure 11(b) shows the AC matching-histogram information on the length information of the data flow.

**Figure 12:**
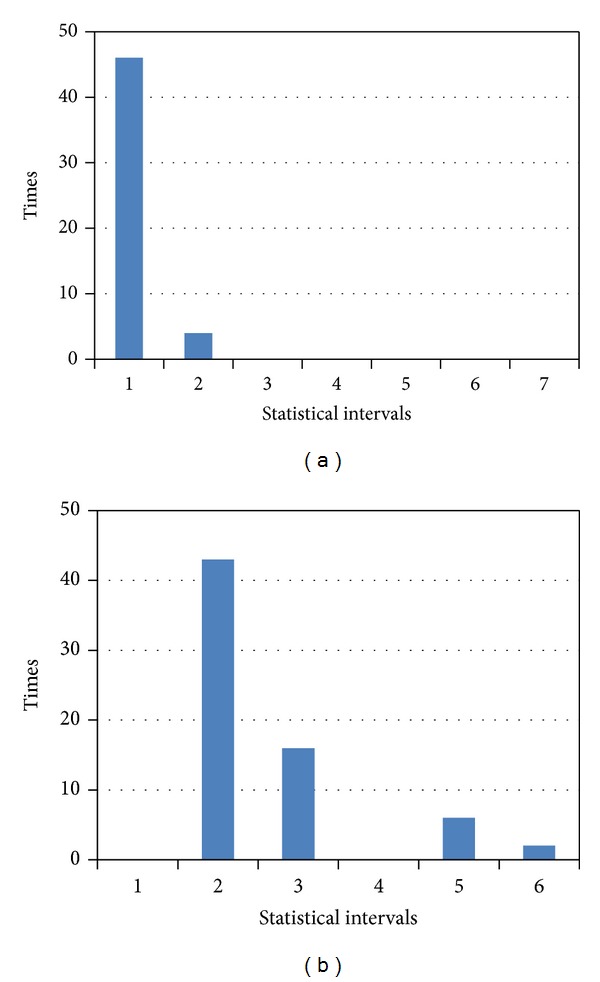
(a) Packet protocol type statistical histogram; (b) TCP control the field statistical histogram information.

Figure 12(a) shows the statistical information of this network traffic according to the partition method described in the previous section.

This indicates that the network traffic mostly consists of TCP packets, as well as a small amount of UDP packets. With respect to the ICMP type and ICMP code information, since the flow does not contain ICMP packets, its statistical information is not recorded.  Figure 12(b) shows the TCP control field information.

**Figure 13:**
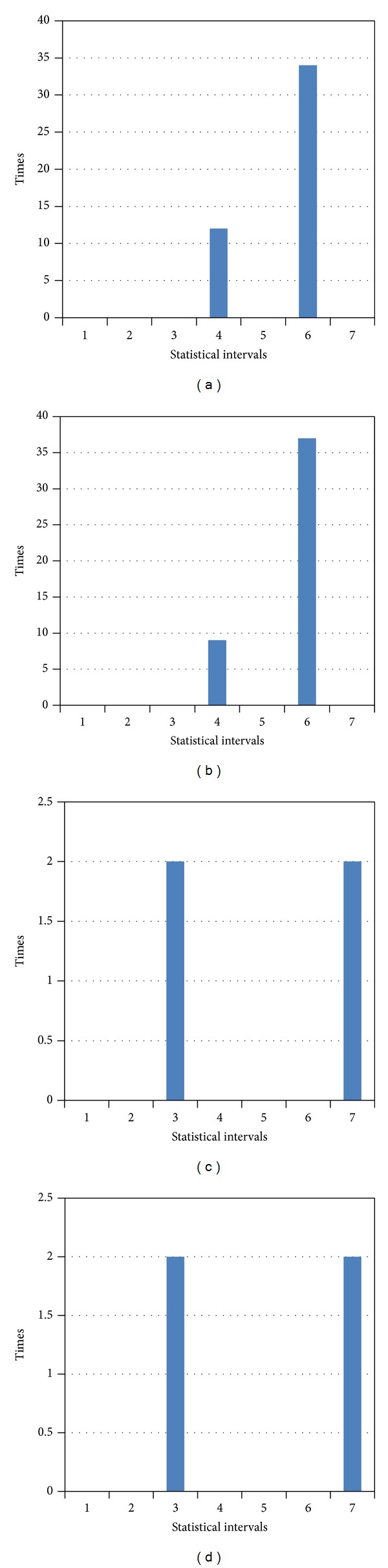
Statistical histogram information of TCP and UDP port number: (a) TCP source port number, (b) TCP destination port number, (c) UDP source port number, and (d) UDP destination port number.

Figures 13(a) and 13(b) show the TCP source and destination port number information according to the previous section. Figures 13(c) and 13(d) show the UDP source and destination port number information.

### 6.5. Matching Classification

After obtaining the statistics on the various characteristics of the data segments, the system will obtain the parameters matching the algorithm required for each network behaviour through repeated training. The investigation established the environment and system and conducted real-time data monitoring and training on three types of instant messaging software, namely, QQ, Fetion, and Renren desktop. In order to enhance the efficiency of the experiment, when the frame length characteristic was extracted, we chose the TCP packet for training and the other protocol type packets were not included in the statistics. The parameters are listed as in Table 6.

Because the system does not capture TCP packets in the training process of the QQ application, the string pattern is empty, so there is no need to conduct matching. And the pattern strings from the Fetion and Renren desktop applications can be seen in Table 7.

Obviously the data packet flows of the Fetion and Renren desktop applications are approximate. However, in comparison with the QQ application, the case is quite different.

The procedures for matching and classification in the following sections are all based on these training data.

### 6.6. System Operation Interface

**Figure 14:**
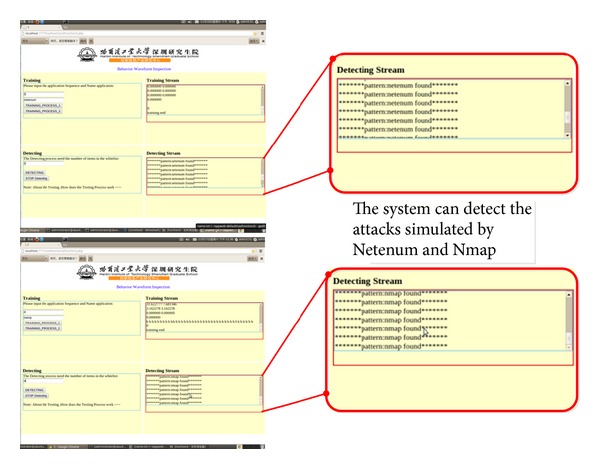
Detection of simulated attacks.

The webpage of the real-time network behaviour detection system designed in this article is shown in Figure 14.

In the training stage, the two edit boxes correspond to the number of training packets and name of the training application which will be processed in the following training steps. The TRAINING_PROCESS_1 button will conduct the extraction process on the features of the training samples, as well as the operation of the establishment of the AC automaton; the TRAINING_PROCESS_2 button will carry out the comparison between the extraction statistics and training data and update the matching thresholds of the various training parameters. Users can conduct training by themselves, as well as increasing the number of training data and patterns according to their needs which indicate the improved scalability of the system.

In the testing stage, the edit box corresponds to the setting of the number of the behavior which will be detected. The DETECTAING button will conduct the real-time detection, and the STOP-DETECTING button will end the detection operation.

Figure 14 shows how to use the system. As can be seen from the figure, when the system is running and after the training stream window outputs each training session, the update of each parameter will be shown and the detecting stream window will display the real-time testing results.

During detection, the system will generate a detection log, to record and prompt the detection of network behavior, as well as the labelled classes and specific data frame number of the network behavior as shown in Figure 14.

In addition we also use two scan tools:* Nmap* and* Netenum*, which are commonly used by hackers, to simulate scan attacks against the gateway. After training, the system can also effectively detect the attacks, which is shown in Figure 14.

In order to check the performance of the system we use the detection system in the laboratory to test 10 network applications. After training, we carried out packet detection on each application and captured 100 groups for each application, a total of 1,000 sets of data. The 10 network applications consisted of Fetion, MSN, Nmap, ooVoo, QQ, the Renren desktop, SkyDrive, Skype, Sina microblogging (Weibo), and World of Warcraft (WoW), and the experimental results are shown in Table 8.

The rows indicate the determination results from the applications, numerical value denotes the situation of accuracy measured, and the columns regard the actual corresponding class of each detected data flow. In case of Renren application, the detection of wrong msn rate is 20/1000 = 2%, miss detection rate is 60/1000 = 6%, and the final detection accuracy rate is 920/1000 = 92%.

Through the above experiment, it can be verified that our system can use the established network behavioural patterns collection to monitor and record the behavior of the application on the user's network, as well as the system's undetected and false detection rates being ideal.

### 6.7. Compared Algorithm-Histogram Statistical Method

The histogram statistical methods will read the frame length information for the data packet and intercept the data frame within a certain time which may represent the characteristics of an attack or an application.

The statistical histogram is counted and generated according to the specific situation. After processing a large amount of data, the statistical interval division will be obtained according to the interval setting strategy in Section 5.

Due to the overbroad numerical range, the histogram distribution is very irregular, which is not favourable for the operator. In order to balance the histogram height, this study has conducted certain interval division of the histogram to obtain more appropriate nonuniform interval segments for calculation. By computing the original histogram of the data again to conduct the second-order histogram statistics, we can obtain the corresponding second order height balanced histogram.

First, obtain the total number of *m* data for the raw histogram information and the ideal histogram interval number *n* after processing. Then, increment the data from the initial value, and accumulate the occurrences of the value *c*_*i*_; that is,

(18) $$\begin{array}{c} m_{i}∶=\overset{i}{\underset{j=0}{\sum }}c_{j}, \end{array}$$

where *m*_*i*_ represents the number of accumulated data which are less than or equal to the value *i*, and then record the value *i* that tends to meet the following conditions as the interval bounds:

(19) $$\begin{array}{c} \frac{m_{i}}{m}∶=\frac{1}{n},\frac{2}{n},\ldots ,1. \end{array}$$

Thus the statistics of each interval can also be obtained by

(20) $$\begin{array}{c} p_{1}∶=\overset{n_{1}}{\underset{i=1}{\sum }}c_{i},\;\;p_{2}∶=\overset{n_{2}}{\underset{i=1}{\sum }}c_{i},\ldots \end{array}$$

So, in this way, it is possible to achieve effective histogram height equalization, for the utilization of the various kinds of arithmetics in the subsequent steps of the system.

After acquiring the histogram statistics, conduct pairwise comparisons and calculate the square of the difference of the two histograms as the basis of classification judgment. Euclidean distance, given by the Pythagorean formula, is used to measure the original distance between two points regulated by some kind of certain rules, and thus the formation of the Euclidean space has also become a metric space [30]. As the most common method of distance calculation, Euclidean distance can be directly and effectively used to reflect differences between objects. In this regard, this method puts forward a measuring algorithm by marking the waveform as the point in the multidimensional space and obtains the degree of matching by measuring the Euclidean distance between the points. This paper treats the points on the waveform as the coordinates of the characteristics in different dimensions, and the length of the waveform is the number of dimensions. In this way, each waveform can be denoted by a point in multidimensional space.

Thus the measurement of the difference between the waveforms is as follows:

(21) $$\begin{array}{c} t=\sum \left(x_{i}^{2}-s_{i}^{2}\right), \end{array}$$

where *t* is the difference, *x*_*i*_ denotes the value of each sample's interval, and *s*_*i*_ means the standard value of each interval. Then repeat the experiments to find the best judgment threshold.

### 6.8. Comparison of Results

#### 6.8.1. Comparison of Three Methods

The histogram method when the matching effect is rough cannot reflect the impact of the time factor on its determination. The AC method is also not sensitive to the time factor, and both of them will generate a large error. However, the AC-histogram method greatly enhances the classification effect.

We selected several instant messaging applications, whose features of the data frames are very close to each other. They are MSN, ooVoo, Paltalk, Skype, and Yahoo messenger. We captured 10 groups of packets for each application and take one of them as the standard data. Then we use different methods of detection on them.

**Figure 15:**
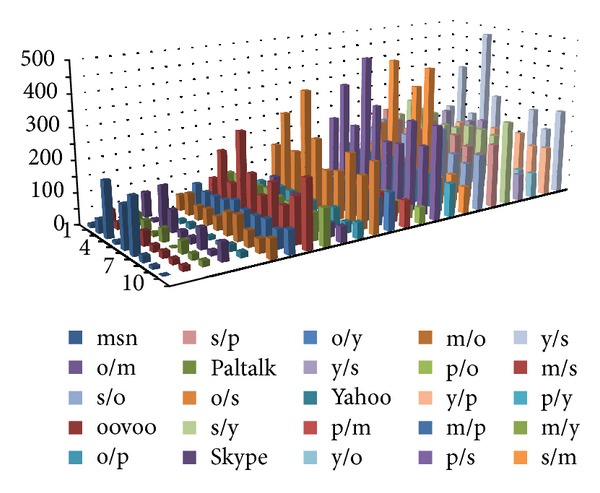
Experimental results of histogram method.

**Figure 16:**
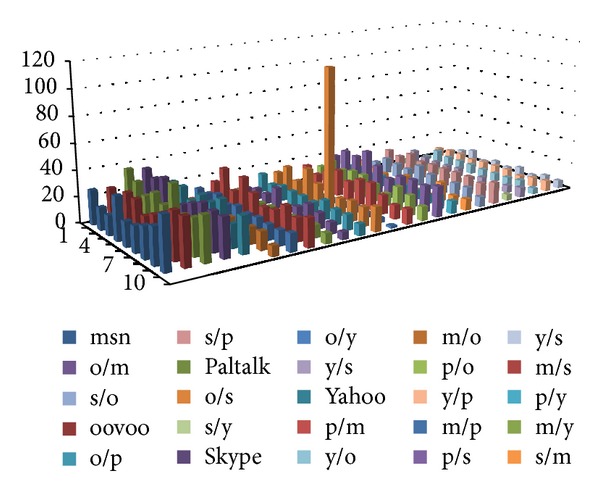
Experimental results of AC method.

**Figure 17:**
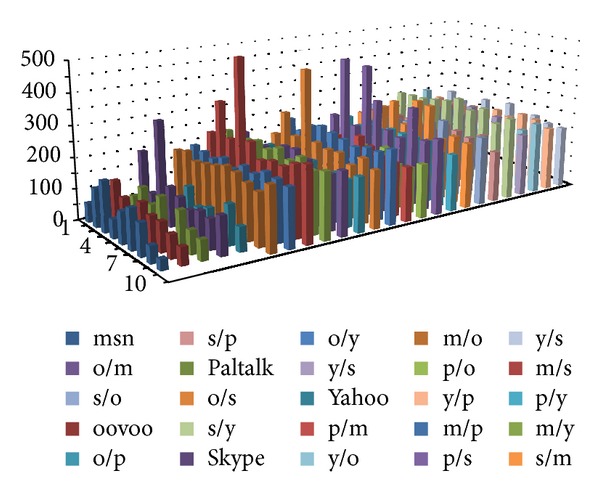
Experimental results of AC-matching and histogram method.

Figures 15, 16, and 17 show the experimental results. The first five rows show the differences between the 10 sets of data for the same category applications. There are 45 sets of data. The remaining rows identify the differences between different applications. There are 200 sets of data. (Simply, m represents MSN, o represents ooVoo, s represents Skype, p represents Paltalk, and *y* represents Yahoo messenger. Thus, m/s denotes the comparison of the experimental results between MSN and Skype, etc.)

#### 6.8.2. Histogram Method

As described in the previous section on the intervals setting strategy, directly conduct statistical processing of the information on data frame length, as well as obtaining statistical histogram information. Then, directly calculate the Euclidean distance between the waveforms which will be regarded as the basis for classification determination.

In order to facilitate the comparison, the calculated distance did not conduct root operations. The experimental results are shown in Figure 15.

The histogram plotted in Figure 16 indicates the accuracy of the matching waveform: when the detecting waveform and the training waveform belong to one class, in fact the same application and the former five cylinders are generally low, which shows the extent of matching accuracy. Indeed, when matching with a different application's waveform, the result will be at a higher value of the distance between the waveforms. However, if the Euclidean distance between the two different application's waveform is also generally low, it is possible to make an incorrect classification.

In order to distinguish between similar applications with heterogeneous applications, we selected the appropriate threshold for the experimental design by a brute-force method to select the optimal threshold for the experiment.

Using the previously mentioned threshold selection method, we selected 28 as the threshold, and the error rate is 16/245 = 6.5%.

#### 6.8.3. AC Waveform Method

Use the proposed algorithm to convert the packet frame length information into a string, and establish the AC automaton for the patterns by the fixed-length substring; then match and record the number of matches for each pattern. Finally, consider the total number of matching pattern strings for statistical test data as the basis for judgment. The experimental results are shown in Figure 16.

As can be seen in Figure 16, when matching with a similar application, the histogram cylinders plotted in the figure are generally high; but when matching with different applications, the number of matching patterns generally shows a low trend.

With previously mentioned threshold selection method, we selected 24 as the threshold, and the error rate is 17/245 = 6.9%.

#### 6.8.4. AC-Matching and Histogram Method

TheAC-matching and histogram method, the advanced algorithm proposed in this paper, similarly uses the Euclidean distance between the waveforms as the basis for classification determination, and in order to facilitate the comparison, the calculated distance also did not conduct root operations. The experimental results are shown below.

First, the system will carry out frame length information quantification and use the AC automaton established in the training stage for pattern matching. Then, the histogram method is conducted to count the number of matches, and the Euclidean distance between the training and testing results obtained from the histogram method procedure is calculated. Thus the similar application shows a lower distance, and different applications show a larger distance. The utilization of the AC-matching and histogram method can get better results in identifying the same application and can also show a high degree of distinction when matching different applications, as can be seen in Figure 17.

By the previously mentioned threshold selection method, we selected 129 as threshold, and the error rate is 5/245 = 2%.

**Figure 18:**
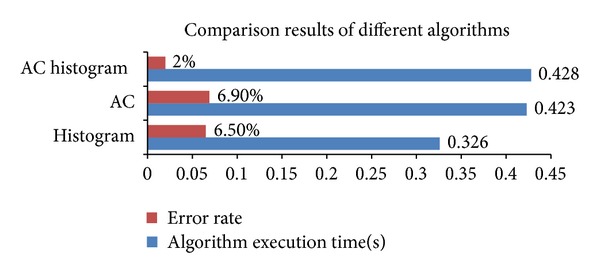
Comparison of different algorithms.

Furthermore, the average execution times and error rates of each method are listed in Table 9. As can be seen from the experimental results, due to the consideration of the prioritization factors of the data packets, as well as the higher matching accuracy (Figure 18), the AC match-histogram method presents the best classification performance. The performances of the histogram method and AC method are close, but the degrees of differentiation are significantly lower than the AC match-histogram method. Furthermore, since the execution time of the AC-histogram method approximates to the two other methods, there is no need to concern the time-consuming problem of the algorithm.

## 7. Conclusion

With Internet and web attacks becoming more frequent and diverse, a new packet detection system with more diverse approaches is needed. In this paper we have constructed a new detection architecture using statistical-based waveforms. In the experiment we appropriately deal with the raw data to obtain the required waveform and then use the classification machine for training and testing.

By using the detection system raised by this study, on the one hand the efficiency of detection can be enhanced, and real-time requirements will be achieved. On the other hand, the concept of a detection whitelist and blacklist is proposed, which overcomes the defects in traditional anomaly detection and misuse detection to some extent. Meanwhile, this study uses statistics within a certain time window as the judgment basis, which increases the accuracy of the determination compared with conventional intrusion detection algorithms based on a single data frame. Compared with complete connection-based intrusion detection, it greatly enhances the convenience of the system and also provides satisfactory judgment accuracy.

## Figures and Tables

**Table 1:** Specific features selected for this paper.

No.	Feature Type	Remark
1	Frame length	Data packet frame length, in bytes
2	Ethernet protocol type	Ethernet type (IP = 0x0800, ARP = 0x0806, RARP = 0x8035, and others recorded as −1)
3	IP protocol type	Such as TCP = 6, UDP = 17, ICMP = 1, and others recorded as −1
4	ARP/RARP opcode	ARP/RARP type of operation (ARP request is 1, the ARP response is 2; RARP request is 3, and RARP response is 4)
5	ICMP type	ICMP message type
6	ICMP code	ICMP message code
7	TCP/UDP source port number	TCP/UDP packet source port
8	TCP/UDP destination port numbers	TCP/UDP packet destination port
9	TCP control field	TCP packet flag field

**Table 2:** Interval setting strategy for frame length.

	Intervals
1	Less than or equal to 50 bytes
2	51–200 bytes (interval of 10 bytes), that is, 51–60 bytes, 61–70 bytes, and so forth
3	201–2000 bytes (interval of 100 bytes), that is, 201–300 bytes, 301–400 bytes, and so forth
4	greater than 2000 bytes

Note: a total of 35 intervals.

**Table 3:** Quantization process for the 9 selected packet features.

Number	Feature type	Values
1	Frame length	The consecutive integer value
2	Ethernet protocol type	2048, 2054, 32832, −1
3	IP protocol type	6, 17, −1
4	TCP/UDP source port number	An integer of 0–65535, −1
5	TCP/UDP destination port numbers	An integer of 0–65535, −1
6	ICMP type	An integer of 0–255, −1
7	ICMP code	An integer of 0–255, −1
8	TCP control field	The consecutive integer value, −1
9	ARP/RARP opcode	1, 2, 3, 4, −1

**Table 4:** Statistics for a piece of network traffic after quantization.

Number	Features number								
	1	2	3	4	5	6	7	8	9
1	60	2048	6	80	55652	−1	−1	18	−1
2	54	2048	6	55652	80	−1	−1	16	−1
3	441	2048	6	55652	55652	−1	−1	24	−1
4	60	2048	6	80	55652	−1	−1	16	−1
5	397	2048	6	80	55652	−1	−1	24	−1
6	54	2048	6	55652	80	−1	−1	16	−1
7	1514	2048	6	80	55652	−1	−1	16	−1
8	54	2048	6	55652	80	−1	−1	16	−1
9	1514	2048	6	80	55652	−1	−1	16	−1
10	54	2048	6	55652	80	−1	−1	16	−1
⋮	⋮	⋮	⋮	⋮	⋮	⋮	⋮	⋮	⋮

Comment: The feature number in this table corresponds to features in Table 4; for example, frame length = 1.

**Table 5:** String pattern.

Short patterns (length 3)								
dqb	qbc	bcq	cqb	qbb	bba	bak	akb	kbb
bad	adb	dbj	bjb	jbe	beb	ebC	bCC	CCC
CCh	Cha	haq	aqb	bbb	baf	afs	fsb	sbC
CCb	Cbb	bbx	bxb	xbp	bpd	pda	daa	aab
abb	bab	dbn						

**Table 6:** Results for three applications after training.

Features	Items					
	QQ		Fetion		Renren desktop	
	Match centre coordinates	Match threshold	Match centre coordinates	Match threshold	Match centre coordinates	Match threshold
Protocol type	(0, 48, 0, 0, 0, 0, 0)	2	(36, 13, 0, 1, 1, 0, 0)	18.44	(32, 15, 0, 1, 0, 0, 0)	22.69
TCP source port number	(0, 0, 0, 0, 0, 0, 0)	0	(0, 0, 17, 0, 0, 6, 19)	11	(0, 0, 8, 0, 0, 0, 33)	19.69
TCP destination port number	(0, 0, 0, 0, 0, 0, 0)	0	(0, 0, 14, 0, 0, 5, 23)	13.93	(0, 0, 9, 0, 0, 0, 32)	19.23
UDP source port number	(0, 0, 0, 0, 0, 48, 0)	4	(0, 0, 0, 0, 0, 3, 0)	3	(0, 0, 0, 0, 0, 3, 0)	3
UDP destination port number	(0, 0, 0, 0, 0, 48, 0)	4	(0, 0, 0, 0, 0, 3, 0)	3	(0, 0, 0, 0, 0, 3, 0)	3
ICMP type	(0, 0, 0, 0)	0	(0, 0, 0, 0)	0	(0, 0, 0, 0)	0
ICMP code	(0, 0, 0, 0, 0)	0	(0, 0, 0, 0, 0)	0	(0, 0, 0, 0, 0)	0
TCP control field	(0, 0, 0, 0, 0)	0	(0, 40, 8, 0, 4, 1)	21.33	(0, 40, 20, 0, 3, 5)	33.31
TCP frame length	—	—	(5, 5, 5, 12, 9, 5)	184	(6, 5, 5, 9, 5, 5, 5)	185

**Table 7:** String patterns of frame length information for three applications.

APP	String patterns for frame length information								
QQ	EMPTY								

Fetion	asB	sBb	BbB	bBa	Bay	ayb	ybb	bba	ban
	ana	nau	auB	uBm	Bmq	mqb	qbb	bbB	bBB
	BBa	BaB	aBB	BBB	Baa	aab	abB	bBy	Bya
	yaw	awr	wrp	Rpb	pbB				

Renren	hbb	bba	ban	anh	nho	hoo	ooa	oap	apf
	pff	ffm	fmw	mwp	wpg	pgp	gpp	ppa	pap
	app	ppn	pnp	npp	ppq	pqm	qmm	mmm	mmp
	mpp	paa	aab	abb	bbp	bps	psr	srb	rbb
	baa	aaq							

**Table 8:** Experimental results from network application detection.

	Fetion	MSN	Nmap	ooVoo	QQ	Renren	SkyDrive	Skype	Weibo	WoW	**miss**
Fetion	100										
MSN		87		7		6					
Nmap			100								
ooVoo		2		93							**5**
QQ					100						
Renren		2				92					**6**
SkyDrive							100				
Skype								100			
Weibo		2		2					91		**5**
WoW										100	

**Table 9:** Average execution times of different algorithms.

Methods	Histogram	AC	AC histogram
Algorithm execution time(s)	0.326	0.423	0.428
Error rate	6.5%	6.9%	2%

## Symbols and Descriptions

AC⁡: Aho-Corasick algorithm
*i*, *j*, *k*: Serial number
*ρ*_*i*_: *i*th character in the text
dist⁡[*ρ*_*i*_]: Distance of the *i*th character needs to jump when match failure occurs in the matching algorithm
*T*: Training
*D*: Detecting
App^(*i*)^: *i*th application
*S*_*T*_^(*i*)^: *i*th training stream
*S*_*D*_^(*j*)^: *j*th testing stream
App_*S*_*T*__^(*i*)^: Label of training stream *S*_*T*_^(*i*)^, which means the training stream truly belongs to App^(*i*)^
App_*S*_*D*__^(*j*)^: Label of testing stream *S*_*D*_^(*j*)^, which is unknown at the beginning
(*S*_*T*_^(*i*)^, App_*S*_*T*__^(*i*)^): Input of training stage
(*S*_*D*_^(*j*)^, App_*S*_*D*__^(*j*)^): Input of testing stage
*L*_*T*_^(*i*)^: Extracted packet frame length information of the *i*th training stream
*L*_*D*_^(*j*)^: Extracted packet frame length information of the *j*th test stream
(*L*_*T*_^(*i*)^, App_*S*_*T*__^(*i*)^): Input sample of data conversion in training stage
(*L*_*D*_^(*j*)^, App_*S*_*T*__^(*j*)^): Input sample of data conversion in testing/detecting stage
*ω*_*T*_^(*i*)^: Generated waveform of training stage for the *i*th application
*ω*_*D*_^(*j*)^: Generated waveform of testing stage for the *j*th application
*δ*[*P*_*m*_]_*T*_^(*i*)^: A string array of group of short patterns generated in the data conversion process of the training stage for the *i*th application
*δ*[*P*_*n*_]_*D*_^(*j*)^: A string array of group of short patterns generated in the data conversion process of the detecting stage for the *j*th application
*ϕ*_*T*_^(*i*)^: Established AC automaton for the *i*th application in the training stage of AC waveform method
*ψ*_*T*_^(*i*)^: Established AC-histogram automaton for the *i*th application in the training stage of the AC waveform and histogram method
*H*_*T*_^(*i*)^: Established histogram for the patterns of the *i*th application in the training stage of the AC Waveform and histogram method
*H*_*D*_^(*j*)^: Established histogram for the matching patterns of the *j*th application in the detecting stage of the AC waveform and histogram method
*C*_*S*_*D*_^(*j*)^_^(*i*)^: Total number of matches of the *j*th detecting stream without application label when inputting it into the *i*th AC automaton, which is only used in the AC waveform method
*P*_*m*_: *m*th short pattern of the training stream with *m* = 1,2, 3,…
*P*_*n*_: *n*th short pattern of the testing stream with *n* = 1,2, 3,…
*C*[*P*_*m*_]_*T*_^(*i*)^: An array to store the number of occurrences of each pattern for the training stream of the *i*th application
*C*[*P*_*n*_]_*S*_*D*_^(*j*)^_^(*i*)^: An array to store the number of matches of each pattern for the detecting stream *S*_*D*_^(*j*)^ when inputting it into the *i*th AC-histogram automaton
*M*_(*j*)_^(*i*)^: Decision-making result of whether the detecting stream *S*_*D*_^(*j*)^ belongs to the *i*th application
*R*_*S*_*D*_^(*j*)^_: Output of the detecting result of the detecting stream *S*_*D*_^(*j*)^
*Va**lu**e*{*condition*(*s*)}: Value that can be obtained if and only if the condition(s) inside the brackets is(are) met
*go**to*( ): goto function of AC algorithm
*fa**il*( ): fail function of AC algorithm
*ou**tp**ut*( ): output function of AC algorithm
Γ_*e*_( ): Preprocessing procedure for data stream, including the extraction of the packet frame length information, and quantification
Λ_*ω*_( ): Waveform generation process for the packet frame length information
Δ_*c*_( ): Process to convert the waveform information into short patterns
Φ_*e*_( ): Establishment of AC automaton
*M*_*c*_( ): Matching process by feeding the detecting data into AC(-histogram) automaton and as a function it will return with a result of true or false
*O*_*c*_( ): Process for counting the number of occurrences of each pattern in the establishment of the AC-histogram automaton
Ψ_*e*_( ): Establishment of the AC-histogram automaton
*H*_*e*_( ): Establishment of the histogram
*d*: Calculation of Euclidean distance
∶ =: Assignment operation
=: Truth assertion
1: Decision-making of matching successful result
0: Decision-making of mismatch result
*m*: Total number of data for raw histogram information
*n*: Ideal histogram interval number after processing
*c*_*i*_: Occurrences of the value
*m*_*i*_: Number of accumulated data which is less than or equal to the value *i*
*p*_*i*_: Statistics of the *i*th interval
*μ*: Consolidated matching value of decision threshold
*μ*_*i*_: Match situation of each feature
*w*_*i*_: Weight of each feature
*t*: Difference between waveforms
*x*_*i*_: Value of each sample's interval
*s*_*i*_: Standard value of each interval.
